# Histogenesis of Merkel Cell Carcinoma: A Comprehensive Review

**DOI:** 10.3389/fonc.2019.00451

**Published:** 2019-06-10

**Authors:** Thibault Kervarrec, Mahtab Samimi, Serge Guyétant, Bhavishya Sarma, Jérémy Chéret, Emmanuelle Blanchard, Patricia Berthon, David Schrama, Roland Houben, Antoine Touzé

**Affiliations:** ^1^Department of Pathology, Centre Hospitalier Universitaire de Tours, Tours, France; ^2^ISP “Biologie des infections à polyomavirus” team, UMR INRA 1282, University of Tours, Tours, France; ^3^Department of Dermatology, Venereology and Allergology, University Hospital Würzburg, Würzburg, Germany; ^4^Departement of Dermatology, Centre Hospitalier Universitaire de Tours, Tours, France; ^5^Monasterium Laboratory, Skin and Hair Research Solutions GmbH, Münster, Germany; ^6^Plateforme IBiSA de Microscopie Electronique, INSERM 1259, Université de Tours, Tours, France

**Keywords:** merkel cell polyomavirus (MCPyV), epithelial, fibroblast, B cell, Merkel cell carcinoma (MCC), histogenesis, origin

## Abstract

Merkel cell carcinoma (MCC) is a primary neuroendocrine carcinoma of the skin. This neoplasia features aggressive behavior, resulting in a 5-year overall survival rate of 40%. In 2008, Feng et al. identified Merkel cell polyomavirus (MCPyV) integration into the host genome as the main event leading to MCC oncogenesis. However, despite identification of this crucial viral oncogenic trigger, the nature of the cell in which MCC oncogenesis occurs is actually unknown. In fact, several hypotheses have been proposed. Despite the large similarity in phenotype features between MCC tumor cells and physiological Merkel cells (MCs), a specialized subpopulation of the epidermis acting as mechanoreceptor of the skin, several points argue against the hypothesis that MCC derives directly from MCs. Alternatively, MCPyV integration could occur in another cell type and induce acquisition of an MC-like phenotype. Accordingly, an epithelial as well as a fibroblastic or B-cell origin of MCC has been proposed mainly based on phenotype similarities shared by MCC and these potential ancestries. The aim of this present review is to provide a comprehensive review of the current knowledge of the histogenesis of MCC.

## Introduction

Merkel cell carcinoma (MCC) is an aggressive neoplasm defined as a primary neuroendocrine carcinoma of the skin. The incidence is still low, with for example 0.7 cases per 100,000 person-years in the United States in 2013, but has increased by 95% from 2000 to 2013, and a further increase in incidence has been predicted (1). MCC occurs essentially in older people, with known risk factors being sun exposure (2) and immunosuppression (3, 4). MCC is characterized by aggressive behavior resulting in a 5-year overall survival rate of 40% (5). Combined radiotherapy and surgery is considered the mainstay of treatment for patients with localized disease, but until recently, those with advanced, inoperable disease received various regimens of cytotoxic chemotherapy, without a significant effect on survival (6). Recently, restoration of T-cell responses by inhibitors targeting programmed cell death 1 (PD-1) and its ligand (PD-L1) checkpoints has been identified as an effective approach in such patients (7). Indeed, after failure of first-line chemotherapy, treatment with avelumab resulted in objective tumoral responses in 32% of MCC patients with advanced disease (7), and avelumab has been approved for advanced MCC both in the United States and European Union (7, 8). Avelumab is being investigated as first-line therapy in this setting, with objective responses in approximately 60% of patients in preliminary reports (9).

**Figure 1:**
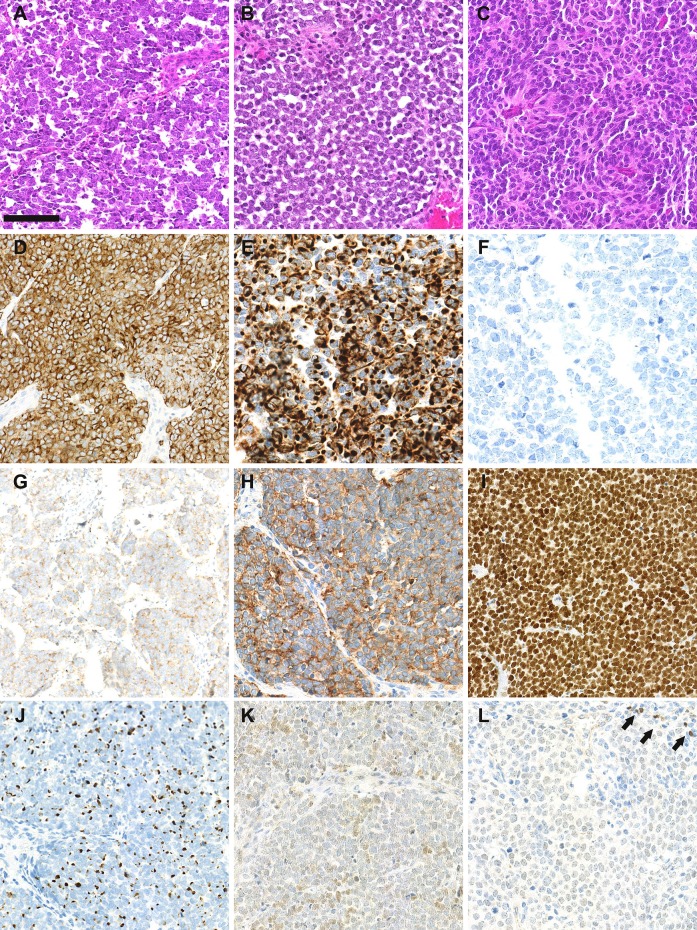
Morphological and immunohistochemical features of Merkel cell carcinoma: **(A–C)**: hematein-phloxin-saffron staining revealed sheet of tumor cells with high mitotic activity (bar = 100 μm). Whereas, MCPyV-positive MCC **(A,B)** harbor scant cytoplasm, round nucleus and dusty chromatin, MCPyV negative tumor cells have more abondant clear cytoplasm and irregular nucleus **(C)**. **(D)** chromogranin A cytoplasmic positivity, **(E)** cytokeratin 20 expression with paranuclear dot-pattern; **(F)** thyroid transcription factor-1 negativity; (**G)** membranous synaptophysin expression; **(H)** membranous CD56 expression; **(I)** special AT-rich sequence-binding protein 2 (SATB2) nuclear expression; **(J)** neurofilament expression with a dot-pattern; **(K)** terminal deoxy nucleotidyl transferase weak/moderate expression, **(L)** paired box 5 weak expression in tumor cells in comparison with intratumor lymphocytes (arrows).

MCC is diagnosed on the basis of histological examination, which reveals infiltration of the dermis or hypodermis by proliferating tumor cells harboring high-grade neuroendocrine carcinoma features (10) (Figure 1). Blastic lymphomas as well as other small round blue cell tumors must be considered in the differential diagnosis. Immunohistochemical investigation of MCC cases (Figure 1) reveals the expression of both epithelial (pancytokeratin AE1/AE3) and neuroendocrine markers such as chromogranin A (11), synaptophysin (11), CD56 (10) and INSM1 (insulinoma-associated 1) (12). In addition, the combination of cytokeratin 20 (CK20) positivity with thyroid transcription factor-1 negativity (13) is currently used to distinguish MCC from other metastatic neuroendocrine carcinomas. Neurofilament and special AT-rich sequence-binding protein 2 (SATB2) have been proposed as additional markers providing high diagnostic accuracy (14, 15).

Significant progress in understanding the MCC pathogenesis occurred in 2008, when Feng et al. reported a yet undescribed virus, the Merkel cell polyomavirus (MCPyV), whose genome was integrated in 80% of MCC tumors (16). MCPyV was further found to be an ubiquitous virus responsible for an asymptomatic life-long infection, because the episomal genome of MCPyV can be detected in the skin flora of most healthy people (17) and antibodies directed against the viral capsid are highly prevalent in the general population (18, 19).

Despite the high population prevalence of MCPyV, viral integration probably occurs very rarely, which accounts for the rarity of MCC tumors, and constitutes the main oncogenetic event leading to MCC oncogenesis. MCPyV integration together with mutations of the viral sequence (20) result in loss of replicative abilities of the virus before MCC development. As a consequence, MCPyV-positive MCC tumors do not produce MCPyV virions but are characterized by permanent nuclear expression of the viral T-antigen proteins (small T [sT] and large T [LT] antigen in a truncated form). Both sT and LT antigens bear oncogenic properties, by targeting various host cell proteins involved in cell cycle control and proliferation, and are now considered as the key actors of oncogenesis in MCPyV-positive MCC (21). By contrast, MCPyV-negative MCC, which accounts for approximately 20% of MCC cases, have a high mutational burdens, with a prominent UV signature, which affects various oncogenes. Among these, mutations of the tumor suppressor genes *RB1* and *TP53* appear to be critical oncogenic events (22).

Despite identification of both viral and UV-induced oncogenetic triggers in MCC, the nature of the cell where MCC oncogenesis occurs remains unknown (23). Actually, several hypotheses have been advanced. The aim of this article is to provide a comprehensive review of current knowledge of the histogenesis of MCC.

### The Merkel Cell: the Historical Candidate

**Figure 2:**
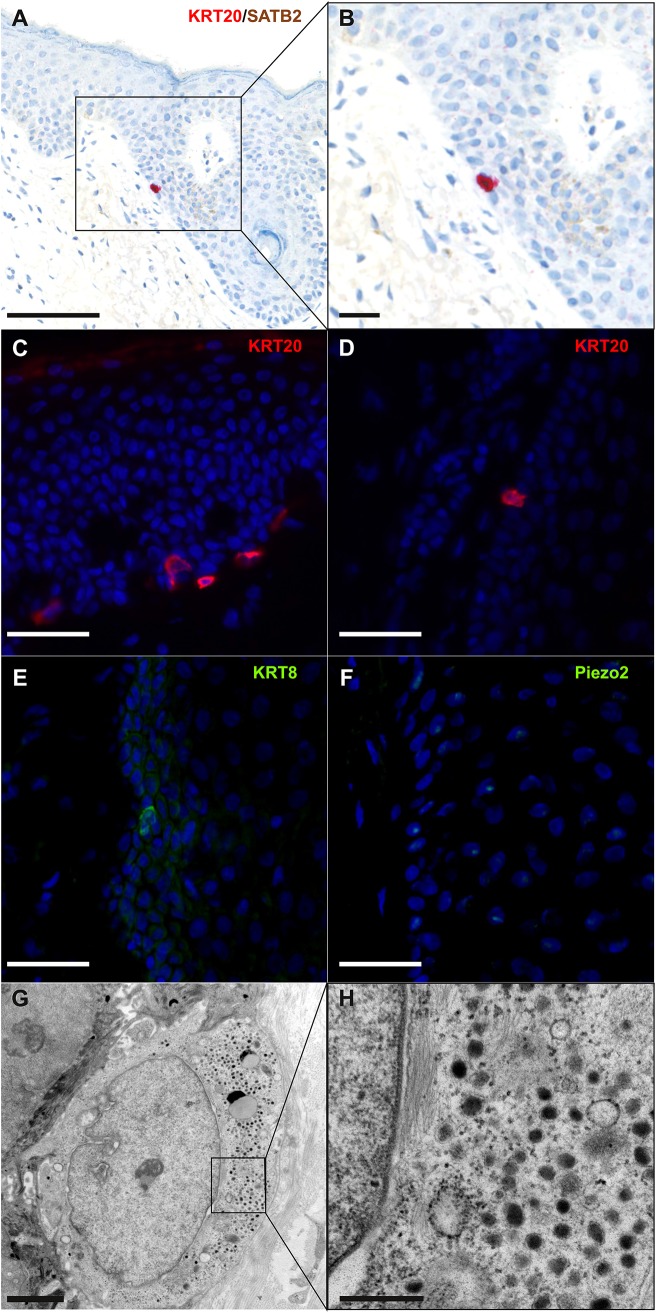
Immunohistochemical and ultrastructural features of physiological Merkel cells: immunohistochemical staining of normal skin **(A,B)** revealed one Merkel cell located in the infundibulum of a hair follicle and coexpressing cytokeratin 20 (cytoplasmic expression in red) and SATB2 (nuclear expression in brown) (bar = 100 and 50 μm for **A,B**). Immunofluorescence staining of healthy skin revealed some Merkel cells expressing cytokeratin 20 **(C,D)**, cytokeratin 8 **(E)** and Piezo2 **(F)** in the epidermis **(C)** and in hair follicles **(D–F)** (bar = 40 μm for **C–F**). Electron microscopy of a Merkel cell **(G,H)** revealed numerous dense-core granules (bars = 2 and 0.5 μm for **G,H**, respectively). A cropped region is shown in the inset **(H)**.

According to Boyd et al. rare cancer types identified before the molecular biology era were “either tumors presumed to originate from or resemble a cell type that infrequently gave rise to cancer, or histologically defined subsets within a more common type of cancer” (24). MCC, a perfect illustration of the first group, was classified according to its similarities with skin physiological Merkel cells (MCs). MCs are highly specialized epithelial cells located in the basal layer of the epidermis and in the external part of the hair follicle (Figure 2). They have been shown to act as mechanoreceptors by transforming tactile stimuli into Ca^2+^-action potentials (25) and serotonin release (26) and pass these signals on to Aβ-afferent nerve endings. The protein allowing transformation of mechanic into electric signals is the ion channel Piezo2 (25), which is also highly expressed by MCC cells [(27), unpublished data]. Expression of this MC-characteristic molecule is only one of many features shared by MCs and MCC cells. Originally described as “trabecular carcinomas of the skin” by Toker (28), ultrastructural studies of such cases revealed numerous neuroendocrine dense cores neuroendocrine granules, which are hallmarks of MCs (28, 29) (Figure 2). Hence, these “trabecular carcinomas” were suggested to derive from MCs, leading to their reclassification as MCC (29). Further immunohistochemical studies corroborated these initial findings by revealing a shared expression of many common markers in MCs and MCC (10, 30) but only a limited number of markers distinguishing them from each other (Table 1; Figures 1, 2). Indeed, both MCs and MCC express cytokeratin 20 (CK20) (13, 15, 31), neuroendocrine markers chromogranin A and synaptophysin (11, 37) and neuropeptides (30, 47). In contrast, the expression of vasoactive intestinal peptide and metenkephalin (44) are specific to MCs, whereas CD117 and CD171 are detected in only MCC cells (49, 61).

**Table 1:** Markers expressed by physiological Merkel cells and Merkel cell carcinoma.

**Markers**	**Merkel cells**	**Merkel cell carcinoma**
**EPITHELIAL MARKERS**		
Cytokeratin 20	+(31, 32)	+(10, 15)
Cytokeratin 8	+(31, 32)	+(33)
Cytokeratin 18	+(31, 32)	+(34, 35)
ß1 integrin	+(36)	
LRIG1	+(36)	
CSPG4	+(36)	
**NEUROENDOCRINE MARKERS**		
Chromogranin A	+(37, 38)	+(10, 11)
Synaptophysin	+(37, 38)	+(10, 11)
CD56	+(39, 40)	+(10, 41)
ISL1	+(42)	+(43)
INSM1	Lacking data	+(12)
Vasoactive intestinal peptide	+(44, 45)	–(44, 45)
Metenkephalin	+(44, 45)	–(44, 45)
MAO A and B	+(46)	Lacking data
**NEUROGENIC/ MECHANORECEPTOR MARKERS**		
Neuropeptides	+(30)	+(47)
Neurofilament	−(48)+	+(14, 15)
CD171	−(49)	+(49)
SATB2	+(50)	+(15, 50)
PIEZO2	+(38)	+(unpublished data)
PGP9.5	+(51)	+(52, 53)
SOX2	+(42)	+(54, 55)
WNT1	+(56)	Lacking data
TUBB3	+(51)	+(57)
p75NTR	+(58)	Lacking data
TrkC	+(58)	Lacking data
NT-3	+(58)	Lacking data
Advillin	+(59)	Lacking data
**B CELL MARKERS**		
CD117 (c-KIT)	–(60)	+(61)
PAX5	Lacking data	+(15, 62, 63)
TDT	Lacking data	+(15, 62, 63)
Immunoglobulins	Lacking data	+(64, 65)

*(+), positivity of the marker; (–), negativity of the marker; CSPG4, chondroitin sulfate proteoglycan 4; INSM1, insulinoma associated 1; ISL1, Islet-1; LRIG1, leucin rich repeats and immunoglobulin like domains 1; MAO, monoamine oxydase; NT-3, neurotrophin 3; p75NTR, neurotrophin receptor p75; PAX5, paired box 5; PGP9.5, ubiquitin C-terminal hydrolase L1; SATB2, special AT- rich sequence binding site 2; SOX2, SRY-box2; TDT, terminal deoxynucleotidyltransferase; TRKC, neurotrophic tyrosine kinase receptor type 3; TUBB3, tubulin beta 3 class III; WNT1, Wnt family member 1*.

Despite the large similarity in phenotypic features, several points argue against MCC deriving directly from MCs. First, in other organs such as lung, strong data suggest that neuroendocrine carcinoma derives more from epithelial progenitors rather than an neuroendocrine cell (66, 67). Second, MCs are mainly post-mitotic cells (31) and thus have low sensitivity to oncogenic stimuli. Accordingly, ectopic expression of sT antigen in MCs failed to induce cell proliferation or transformation in a transgenic mouse model (68). Of note, hyperplasia of MCs as well as mitotic activity in keratin 20-positive cells has been reported in pathologic conditions (69, 70); however, whether these observations are due to proliferation of already differentiated MCs or MC precursor cells is still unclear. Third, MCs are most frequently present in the palm and sole in humans (71, 72), whereas MCC occurs mainly in sun-exposed areas [head and neck, legs (2, 73)]. Moreover, no infection of MCs by MCPyV has been reported (74). Finally, in an *in vitro* model, MCPyV pseudovirions could barely infect CK20-positive cells obtained from the fetal scalp (0.8%) (75), which argues against an efficient MCPyV infection triggering MCC oncogenesis in an already differentiated MC.

### Putative Mechanism of a “Non-MC” Origin for MCC

The tumor classification system is based on tumor differentiation and should not be considered a direct indicator of tumor histogenesis (76). Indeed, several phenotypic changes occurring during the oncogenic process contribute to the final differentiation profile of tumor cells, which consequently differ from the primary cell in which the first oncogenic event took place (76). Accordingly, acquisition of an MC-like phenotype including neuroendocrine differentiation (77) during MCC oncogenesis could explain the similarities between MCs and MCC (23). In MCC, both UV and virus-induced oncogenic triggers are thought to act on shared molecular pathways, accounting for the similar phenotype between MCPyV-positive and -negative tumors (78). In this respect, disruption of pRB function occurs by somatic mutations and repression of protein expression in virus-negative tumors (22), whereas sequestration by MCPyV LT antigen inactivates pRB1 in virus-positive MCC cells (79). Interestingly, disruption of this pathway has been identified as a main contributor driving acquisition of a neuroendocrine phenotype in tumors of other organs (80–82).

In the skin, MC differentiation occurs in specific epithelial precursors upon expression of one main transcription factor, atonal homolog 1 (ATOH1) (31). Under physiologic conditions, ATOH1 expression in the skin is restricted to MCs (31). Because ATOH1 is also observed in MCC, its expression could explain the shared phenotype between MCs and MCC (83). Moreover, genetic ablation of Rb1 and the related Rb-family protein p130 in the intestinal epithelium in a mouse model led to increased expression of Atoh1 (84), which suggests that Atoh1 induction could occur during an oncogenic process associated with Rb inactivation.

**Figure 3:**
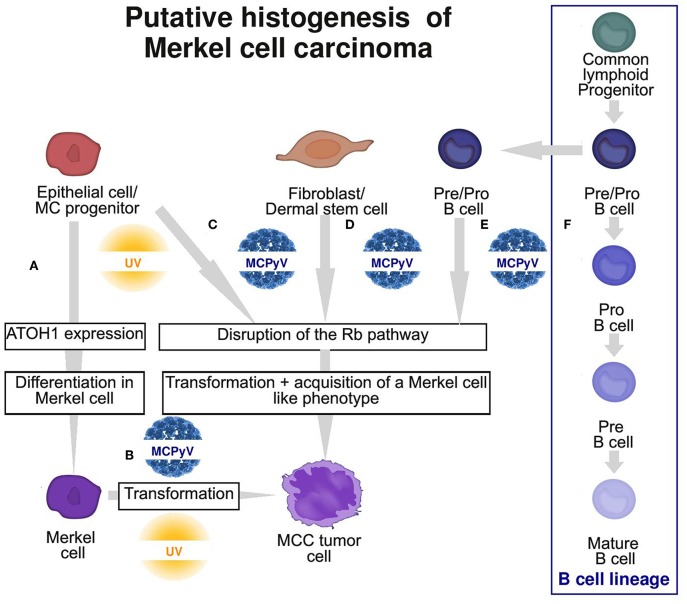
Graphic summary of the 4 putative cells of origin of Merkel cell carcinoma (MCC). **(A)** Physiological MC differentiation **(B)** First hypothesis: physiological MC as the cell of origin of MCC, suggesting that T antigens can induce transformation in this cell type. **(C–E)** Second hypothesis: oncogenic events occur in a non-MC and induce transformation and acquisition of an MC-like phenotype. Potential ancestries are epithelial progenitors **(C)**, fibroblast/dermal stem cells **(D)** or pre/pro B cells **(E)** from the B cell lineage **(F)**. MC, Merkel cell; MCPyV, Merkel cell polyomavirus.

Considering these findings, a non-MC could also be candidate for the ancestry of MCC, and an epithelial non-MC as well as a fibroblastic and B-cell origin has been proposed (Figure 3; Table 2).

**Table 2:** Pros and cons of current hypotheses for the potential cell of origin of Merkel cell carcinoma (MCC).

**Candidate**	**Pros**	**Cons**
Merkel cell	Phenotypic similarities: (immunohistochemical profile: CK8, CK18, CK20 + neuroendocrine markers+ultrastructural findings)	No mitotic activity No demonstrated MCPyV demonstration No transformation by MCPyV antigens Lack of epidermal connection in almost all MCC cases
Epithelial progenitor	Ability to differentiate into Merkel cells Ability to generate combined MCC Most probable origin of neuroendocrine carcinoma in other sites	Exclusive dermal/hypodermal location of MCC No UV signature Lack of epidermal connection in almost all MCC cases
Fibroblast and dermal stem cell	Site of replication of the MCPyV Ability of MCPyV antigens to induce transformation in these cell types Presence of SKP with reprogramming abilities	No proof of the ability of fibroblasts to acquire an MC-like phenotype Unexpected origin for a neuroendocrine carcinoma
Pre/pro B cell	Epidemiologic association between MCC and B-cell neoplasia Co-expression of B-cell markers (PAX5, TdT and Immunoglobulins) Detection of MCPyV integration in B-cell neoplasia	No proof of the ability of B cells to acquire an MC-like phenotype Unexpected origin for a neuroendocrine carcinoma

*MC, Merkel cell; MCPyV, Merkel cell polyomavirus; SKP, skin-derived precursors*.

### A Non-MC Epithelial Origin

For quite some time it has been a matter to debate whether MCs derive from the neural crest or epidermal lineage. Of note both neural crest and epidermal lineages derived from the same embryologic structure and this common ectodermal origin might explain the mixed phenotype observed in Merkel cell Indeed, ultrastructural studies of MC revealed on the one hand intracytoplasmic neuroendocrine granules suggesting a neural crest origin (85) and on the other hand frequent desmosomes and cytokeratins, two hallmarks of the epithelial subset (86). Accordingly, also immunohistochemistry demonstrated both expression of “neural crest” as well as epithelial markers (Table 1). Although the neural crest origin hypothesis was additionally supported by chimeric chicken/quail models (87, 88), xenograft of human fetal skin free of neural crest progenitors in immunocompromised mice led to the development of human Merkel cell suggesting an epidermal origin of this population (89).

An epithelial origin of Merkel cells in mammals was finally demonstrated in 2009 by two consecutive transgenic mouse studies (31, 90). In both studies it was shown that deletion of Atoh1 in epidermal progenitors resulted in a complete absence of MCs. Additionally, Morrison and colleagues demonstrated that Atoh1 deletion in the neural crest lineage did not affect the MC population (90).

Additional studies in mice models revealed that MC phenotype acquisition upon Atoh1 expression seems to be restricted to a specific subpopulation of keratinocyte progenitors characterized by an activated Sonic Hedgehog pathway (91, 92). Indeed, Atoh1 expression failed to induce MC differentiation in other keratinocyte populations (31) and gave rise to distinct differentiation in other cell types (93–95).

A thorough characterization of the MC progenitor population in humans is still missing (96). Therefore, our current knowledge of this cellular subset is mainly based on findings in mice, in which cells bearing MC differentiation potential are mainly located in the outer root sheet and bulge region of the hair follicle (97, 98) but are also present in the interfollicular epidermis in specialized structures called touch domes (92). Interestingly, these hair follicle- and touch- dome–derived stem cells have been found as preferentially the origin of basal cell carcinomas (99). Therefore, their ability to acquire an MC phenotype and to proliferate, as well as their high sensitivity to oncogenic stimuli, should promote their transformation into MCC, rendering them likely candidates as cells of origin. Of note, MCC developing within follicular cysts (100) as well as preferential MCPyV infection of the dermal cells around hair follicles (75) support MCPyV(+) MCC as being derived from hair follicles.

A hair-follicle origin of MCC would also weaken one argument frequently used against an epithelial origin of MCC. Because MCC cells are mostly found in the dermis and subcutis lacking a connection to the epidermis, an epidermal origin is unlikely (62). However, some appendage tumors such as trichoblastoma and spiradenoma (101, 102) are well known to lack an epidermal connection (10).

**Figure 4:**
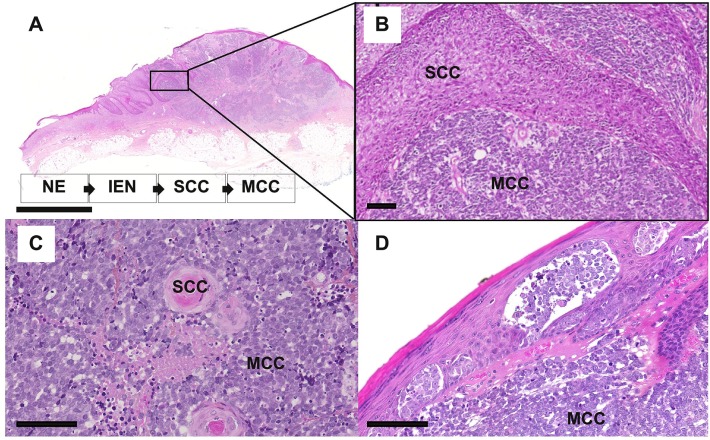
Microscopy features of MCC with divergent differentiation or intra-epidermal involvement [bars = 5 mm and 200 μm **(A,B)** and 100 μm **(C,D)**. **(A–C)** combined MCC is characterized by the association of MCC with another differentiation subset, mainly squamous cell carcinoma (SCC). In some specimens, intra-epidermal neoplasia (IEN) such as Bowen disease, deriving from the non-neoplasic epidermis (NE) can be detected in tumor in close contact. **(D)** MCC harboring an intra-epidermal component.

The observation of so-called combined MCC or MCC with divergent differentiation further supports an epithelial origin of MCC. Combined MCC represents 5 to 10% of cases and is characterized by the association of an MCC component with a tumor of another differentiation lineage (103–105). Although several divergent additional components have been described (sarcomatous, adnexal) (104, 106), MCC is most frequently found associated with squamous/eccrine carcinoma (105, 107) (Figure 4). For individual cases, the same genetic alterations have been reported for both components, which implies a common progenitor (108), whereas other cases gave proof of a collision tumor (109). Furthermore, similar aberrant p53 expression is frequently observed in both components of combined MCC (105). In some combined MCC cases, intra-epidermal neoplasia such as actinic keratosis or Bowen's disease (107) was detected close to the squamous cell carcinoma component. Bowen's disease originates from the epidermis, and invasive squamous cell carcinoma can derive from Bowen's disease; hence, the clonality between squamous cell carcinoma and the MCC component (108) favors an epidermal origin of MCC (97). Of note, the hyperplasia of MCs in the squamous cell carcinoma component of combined tumors (70) might suggest that such components contain precursors with the ability to acquire an MC phenotype.

Importantly, such combined cases have been described to be usually typical UV-induced MCCs, harboring morphologic and immunohistochemical features distinct from MCPyV-positive MCC and high mutational load (104, 106, 108) as depicted in Table 3. Of note low viral load of MCPyV in some cases is probably related to an episomal viral genome present in the skin (105). In our experience [(118), Figure 4], rare cases of MCC with intra-epidermal involvement [2% in our previously reported cohort (73)] are also related to the UV-induced subset. Hence, although combined cases imply that MCPyV-negative cases derive from some epidermal progenitors of the interfollicular epidermis, they provide no information about MCPyV-induced tumors (119).

**Table 3:** Distinct features of MCPyV-positive and -negative MCC cases.

**Features**	**MCPyV(+) Merkel cell carcinoma**	**MCPyV(–) Merkel cell carcinoma**
**MORPHOLOGY**		
Nucleus	Round (110, 111)	Irregular/spindle (110, 111)
Cytoplasm	Few (110, 111)	More abundant (110, 111)
Divergent differentiation	No (103, 104)	Yes (103, 104)
**IMMUNOHISTOCHEMICAL MARKERS**		
CK20	+(112, 113)	+/–(112, 113)
CK7	–(112)	+/–(112)
TTF1	–(112, 114)	+/–(112, 114)
Neurofilament	+(14, 106, 112)	+/–(14, 106, 112)
Oncogenic triggers	MCPyV T antigens (16, 68, 79, 115)	UV induced genetic alteration (22, 116, 117)
Mutation load	Low (22, 116, 117)	High (22, 116, 117)

*(+), frequent positivity of the marker; (–), frequent negativity of the marker; (+/–) increased or decrease expression frequency of this marker compared to the MCPyV(+) subset*.

*Compared to the MCPyV-positive MCC cells MCPyV-negative MCC tumor cells have been described to harbor more irregular nuclei, more abundant cytoplasm and display more frequently so called divergent differentiation. Moreover, MCPyV-negative cases are characterized by an specific immunohistochemical profile with frequent lack of expression of CK20 and neurofilaments, and more frequent positivity for TTF1 and CK7. Finally, very high mutational burden with UV signature are observed only in MCPyV-negative cases*.

In agreement with this observation, Sunshine et al. hypothesized that there might be two different cells of origin for the two MCC subtypes (119). They provided several arguments for this conclusion. For example while the UV-mutation signature of virus-negative MCC favors an epidermal origin the failure of epidermis targeted TA-expression to produce tumors resembling human MCC in mouse models (68, 120, 121) suggests that other cells in the skin such as dermal fibroblast may serve as origin of MCC (119). Since both UV- and virus-induced MCC occur in sun-exposed areas where frequent UV-induced mutations are observed in keratinocytes (122), but only MCPyV-negative cases are characterized by high mutational load and UV signature (22, 119) Sunshine and colleagues excluded an epithelial and instead proposed a fibroblastic origin of MCPyV(+) MCC (119). However, low mutational burden as well as lack of UV-signature in MCPyV(+) MCC might also be explained by MCPyV integration into a cell from the hair follicle which like dermal fibroblasts is located deeper in the skin then normal epidermal keratinocytes.

In conclusion and acting on the assumption that MCC generally has an epithelial origin, one could speculate that UV-induced MCC derives from a keratinocytic progenitor from the interfollicular epidermis that acquires the ability to differentiate into MCs during the oncogenic process, whereas MCPyV-driven oncogenesis is initiated in a progenitor from a hair follicle.

### A Fibroblastic Origin

Another hypothesis is MCC developing from fibroblastic cells. This hypothesis might account for the quasi-exclusive dermal location of MCC, discussed above. Furthermore, the fibroblastic origin of MCCs would be consistent with our knowledge of the MCPyV cycle because fibroblasts of the papillary dermis have been identified as the main site of replicative MCPyV infection (75). Although infectious MCPyV particles can enter several cell types including keratinocytes with various efficiency rates (75, 123), fibroblasts remain the only host cell evidencing early and late viral protein expression. One could argue that replication and transformation can occur in independent cell types, as was previously demonstrated for polyomavirus SV40 (124); however, the ability of fibroblasts to allow replication of the MCPyV genome increases the likelihood of accidental integration of the viral genome. Moreover, the *in vitro* transforming potential of sT antigen has until now been demonstrated only in fibroblasts (68, 124, 125). Notably, ectopic expression of SV40 T antigens in fibroblastic cells (126) triggered the induction of cytokeratin expression, which suggests that polyomavirus infection can influence a differentiation lineage. In such a setting, acquisition of an MCC phenotype induced by viral protein expression could require a transient pluripotent stage. Indeed, fibroblasts are widely used for reprogramming to pluripotent cells. The resulting induced pluripotent stem cells (127) can be differentiated into epithelial cells *in vitro*. Furthermore, physiological stem cells of the papillar dermis [i.e., dermal skin precursors or skin-derived precursors (128)] share phenotypic similarities with induced pluripotent stem cells, such as expression of the stem cell factors c-Myc and Sox2 (129), two markers also expressed by MCC (54, 130). These dermal skin precursors are able to differentiate into epithelial or neuronal cells *in vitro*. Hence, because of the close proximity of these cells to dermal fibroblasts, which can support productive MCPyV infection (75), as well as their expression of pluripotent factors and their differentiation abilities, MCPyV integration in such cells could lead to MCC oncogenesis and acquisition of an MCC phenotype.

### A Pre/Pro or Pre–B-Cell Origin

Because of the recurrent association between MCC and B-cell neoplasias (131–134) as well as phenotypic similarities and the occasional integration of MCPyV in hematopoietic cells, a lymphoid pre/pro B-cell origin is also discussed (62, 64).

Indeed, chronic lymphocytic leukemia is the most frequent neoplasia associated with MCC development. Whether this is due to a common transforming event or the first tumor creating an immunological microenvironment facilitating the development of the second tumor or merely due to both tumors appearing in older immunocompromised subject has yet to be determined (131).

Moreover, MCC shares morphological features with other small round blue cell tumors, which explains why B-cell neoplasia must be considered a differential diagnosis of MCC. In addition, the coexpression of terminal deoxy nucleotidyl transferase (TdT), paired box 5 (Pax5) and immunoglobulin chains, all markers expressed during B-cell differentiation, has been observed in MCC tumors (62, 64). Initially, the frequency of TdT and Pax5 positivity was reported to be about 65% (*N* = 187) and 90% (*N* = 143) of MCC cases (64); however, recently observed rates were lower, 26% (*N* = 217) or 23% (*N* = 213) (15, 63). Of note, expression of immunoglobulin chains was restricted to the MCPyV(+) subset and detected in about 70% of cases (65). In addition, rare observations of MCC cases with monoclonal immunoglobulin rearrangement of heavy chain as well as monoclonal expression of Kappa light chain were reported (62, 65). As already discussed, determination of the histogenesis based on phenotype similarities between terminally differentiated tumor and physiological cells does not account for phenotypic changes during oncogenesis (76). In this regard, induction of immunoglobulin expression during the oncogenic process has been reported for several epithelial and soft-tissue neoplasias (135, 136) and may contribute to tumor aggressiveness (137). Furthermore, immunoglobulin rearrangement due to the expression of essential enzymes required for gene rearrangement and class switch recombination has been described in non-hematopoietic neoplasia (136). Hence, immunoglobulin expression and rearrangement might result from the oncogenic process, and their occurrence in MCC cannot rule out a non-lymphoid cell origin. Induction of immunoglobulin expression in epithelial cells has been reported to result from Epstein-Barr virus infection (138) and was also observed in papillomavirus-induced neoplasia (139). These findings, combined with the exclusive expression of immunoglobulins in MCPyV(+) MCC, led Murakami and colleagues to hypothesize that the immunoglobulin expression in MCC cells is induced by MCPyV oncoproteins (65). In the same manner, the concomitant expression of TdT and Pax5 is restricted to immature B cells and thymocytes under physiological conditions (140) and is also observed in MCC. While co-expression have not yet been described positivity of one of these markers has also been demonstrated in several epithelial neoplasias (141, 142), which indicates that these markers can be acquired during the oncogenic process. Moreover, MCPyV genome integration (143) associated with a deletion leading to a truncated LT antigen (144), the two hallmarks of MCC oncogenesis, have been evidenced in some cases of chronic lymphocytic leukemia and tropism of other tumor viruses for the Pre-Pro B cells has been previously emphasized (145). Although these findings demonstrate that MCPyV integration associated with transformation can occur in B cells, lack of acquisition of an MCC phenotype in these cases argue against a B-cell origin of MCC.

## Summary

To conclude, reviewing the current knowledge of MCC histogenesis allows for also underlining the basis of the current tumor classification system. Indeed, tumors are mostly classified according to their differentiation status and their level of similarities with physiological cells at the same location (24). However, we should keep in mind that the final phenotype of a given tumor cell may result from strong differentiation changes occurring during oncogenesis and thus does not necessarily directly reflect the cell ancestry (76). Accordingly, despite strong similarities, MCC likely does not derive from already differentiated MCs, which suggests that acquisition of an MC-like phenotype occurs during the oncogenic process (Figure 3). From the observations of combined MCC tumors, high somatic pathologic variant loads and detection of an UV signature in this subset, UV-induced MCC cases probably derive from a progenitor cell of the epidermis. By contrast, the nature of the cell in which MCPyV integration occurs remains to be clarified. The lack of connection between tumor cells and the epidermis as well as lack of a UV signature could favor a non-epithelial origin but alternatively could be explained by integration of MCPyV in cutaneous appendage enriched with MC precursors. Use of experimental models in addition to phenotypic characterization of MCC to monitor phenotype changes induced by MCPyV in several cell types are needed to fully address this question.

## Author Contributions

All authors listed have made a substantial, direct and intellectual contribution to the work, and approved it for publication.

### Conflict of Interest Statement

The authors declare that the research was conducted in the absence of any commercial or financial relationships that could be construed as a potential conflict of interest.
